# Exploring the genomic landscape of Chlamydiifrater species: novel features include multiple truncated major outer membrane proteins, unique genes and chlamydial plasticity zone orthologs

**DOI:** 10.1099/acmi.0.000936.v3

**Published:** 2025-02-03

**Authors:** Martin Hölzer, Charlotte Reuschel, Fabien Vorimore, Karine Laroucau, Konrad Sachse

**Affiliations:** 1 Bioinformatics and Translational Research, Genome Competence Center, Robert Koch Institute, 13353 Berlin, Germany; 2 Laboratory for Animal Health, Identypath, ANSES Maisons-Alfort, Paris-Est University, 94706 Paris, France; 3 Laboratory for Animal Health, Bacterial Zoonosis Unit, ANSES Maisons-Alfort, Paris-Est University, 94706 Paris, France; 4 RNA Bioinformatics and High-Throughput Analysis, Friedrich Schiller University Jena, 07743 Jena, Germany

**Keywords:** *Chlamydiifrater phoenicopteri*, *Chlamydiifrater volucris*, *Chlamydiifrater*, genome analysis, *ompA *family proteins, re-annotation strategies

## Abstract

Recently discovered obligate intracellular bacteria belonging to the genus *Chlamydiifrater* with the species of *Chlamydiifrater phoenicopteri* and *Chlamydiifrater volucris* were studied to explore the composition of their genomes and their relatedness to *Chlamydia*, the other genus of the family *Chlamydiaceae*. We investigated 4 isolates of *Cf. volucris*, 2 of them newly sequenced, and one of *Cf. phoenicopteri* alongside 12 representatives of the *Chlamydia* species. Our study uncovers previously unrecognized genomic structures within *Chlamydiifrater* using a hybrid sequencing approach and advanced annotation pipelines, providing insights into species-specific adaptations and evolutionary dynamics. The integration of long-read sequencing data, comprehensive re-annotation strategies and pan-genomics enabled the localization of the unique plasticity zone and the identification of novel gene clusters in *Chlamydiifrater* strains, which improves our understanding of chlamydial genome architecture and plasticity in the family *Chlamydiaceae*. Our analysis revealed that 761 CDS (~80%) are shared among members of both genera. We further identified 158 unique genes of *Chlamydiifrater* species, but their annotation remains challenging because of the absence of functionally annotated orthologs in public databases. A full-length *ompA* gene encoding the major outer membrane porin was seen in all *Chlamydiifrater* strains. We also describe the localization and structure of multiple truncated CDS of *ompA* family members, representing one of this study’s most interesting findings. While genome analysis of *Chlamydiifrater* spp. confirmed numerous common features shared with representatives of the genus *Chlamydia,* many unique genomic elements were identified that underpin the distinct phenotype and separate genetic position of these new microorganisms.

**Impact statement**

We present two new high-quality genome sequences of *Chlamydiifrater* strains and conduct a comprehensive genome analysis of five *Chlamydiifrater* genomes. Combining Illumina and Nanopore sequencing data and using our own annotation pipeline, we revealed common features and specific distinctions from the closest relatives, *Chlamydia* spp. These findings will help to understand the biology and infection mechanisms of these recently discovered bacteria.

## Data Summary

The Illumina and Nanopore sequencing data produced for two *Chlamydiifrater* strains during this study were uploaded to ENA under accession PRJEB59173 comprising raw reads for strain O77 (Nanopore, ERR10829098; Illumina, ERR10829101) and strain O09 (Nanopore, ERR10829099; Illumina, ERR10829100). All selected input genomes, their re-annotations, all intermediate analysis files and the pangenome calculations via RIBAP are deposited in the Open Science Framework under accession https://osf.io/dtwej. Two supplementary tables are available with the online version of this article.

## Introduction

Bacteria of the family *Chlamydiaceae* are defined as ‘coccoid, non-motile, obligate intracellular organisms of 0.2 to 1.5 µm diameter that reside in vacuole-like inclusions of eukaryotic cells, where they parasitize and multiply in a unique developmental cycle’ [1]. The cycle initiates as small elementary bodies (EBs) invade the host cell through distinct pathways involving diverse receptor molecules. Once inside, EBs induce the formation of a vacuole-like inclusion, where they differentiate into larger reticulate bodies (RBs) and multiply by binary fission. Finally, RB-to-EB reversion is accompanied by inclusion rupture, extrusion and/or formation of *Chlamydia*-containing spheres [2], leading to the release of new infectious EBs. *Chlamydiaceae* spp. depend on eukaryotic cells for survival and replication, which presents a significant challenge for culturing and *in vitro* studies. Initially, chlamydial strains were isolated using embryonated chicken eggs, but later, cell culture became the preferred technique in research and diagnostics.

In the past, all species within the *Chlamydiaceae* family were classified under just one genus, *Chlamydia* [1]. This genus comprises 18 species, with 4 recognized under the *Candidatus* classification. In a previous paper, we described three new strains isolated from clinically inapparent flamingos in various regions of France and showed that they belong to a separate genus named *Chlamydiifrater* [3]. Through an analysis of ribosomal RNA sequences, average nucleotide identity, the percentage of conserved proteins, tetranucleotide signature correlations, nine conserved proteins and various genomic parameters, we confirmed the genetic position of *Chlamydiifrater* being outside the *Chlamydia* genus. We proposed the classification of two new species: *Chlamydiifrater phoenicopteri* and *Chlamydiifrater volucris*. While the *Chlamydiifrater* phenotype contains many of the typical characteristics known from *Chlamydia* and other obligate intracellular bacteria, transmission electron microscopy revealed a different inclusion morphology in host cells with multiple confluent inclusions, thin strands of cytoplasm between them and the apparent absence of intermediate bodies alongside EB and RB [3]. These distinctive features could indicate differences in certain stages of pathogen–host interaction. However, as *Chlamydiifrater* organisms were discovered only recently, future studies need to address these aspects. Likewise, data on potential pathogenicity and epidemiology have yet to be obtained.

Our previous paper also contained preliminary data on the genome composition of the new *Chlamydiifrater* species. An initial examination of whole-genome sequences indicated that *Chlamydiifrater* strains have about 75% of their genomes in common with members of the genus *Chlamydia* [3]. On the other hand, we also found a considerable portion of the genome that occurred only in *Chlamydiifrater* strains. A distinctive genomic feature identified was the presence of several truncated *ompA*-like gene sequences in addition to the full-length *ompA*, which encodes the major outer membrane porin (MOMP) [3]. Consequently, the question arose whether typical genome elements known from *Chlamydia* spp., such as *pmp* and *inc* gene families, plasticity zone (PZ) and putative virulence factors were also present in *Chlamydiifrater* and, if so, how they compared to their counterparts in the genus *Chlamydia*. Therefore, we undertook a more comprehensive comparative study, presented below.

So far, only three strains of *Chlamydiifrater* spp. have been preliminarily described and analysed. We cultured and sequenced more strains to explore the new species further. At the same time, we improved genome analysis by combining Illumina and Nanopore sequencing data (a hybrid approach) and using our recently developed RIBAP tool for pangenome calculations beyond the species level [4].

The present study was undertaken to characterize *Chlamydiifrater* genomes in more detail, including sequences of new isolates, identifying genomic properties that distinguish them from *Chlamydia* spp. and highlighting the unique features of these recently defined bacteria.

## Methods

### Bacterial strains

The 17 chlamydial strains included in this study and the sources of whole-genome sequences are listed in Table 1. Under the given accession numbers, the FASTA and GenBank annotation files were downloaded from the National Center for Biotechnology Information (NCBI) database. The genomes of *Waddlia chondrophila* 86–1044 and *Simkania negevensis* Z were taken as additional outgroups for phylogenetic investigations from GenBank entries NC_014225.1 and NC_015713.1, respectively. The obtained GenBank annotation files were used to guide the re-annotation via Prokka (Table S1, available in the online version of this manuscript). All 19 input genomes (19 FASTA files, 17 GenBank files used for re-annotation) and further intermediate and final result files were uploaded to the online repository of the Open Science Framework (https://osf.io/dtwej).

**Table 1.** Basic genomic parameters of the strains analysed in this study

Species strain	Chromosome length (bp)	CDS	GC (mol-%)	5S, 16S, 23S rRNA	tRNA	tmRNA	Plasmid length (bp)	Chromosome NCBI acc. no.
*Cf. phoenicopteri* 14–2711_R47	1 195 517	981	40.30	1, 1, 1	39	1	6358	NZ_LR777658.1
*Cf. volucris* 15–2067_O50	1 197 353	979	40.42	1, 1, 1	38	1	6320	NZ_LR777654.1
*Cf. volucris* 15–2067_O99	1 198 895	982	40.41	1, 1, 1	38	1	6319	NZ_LR777656.1
New isolate 15–2067_O09	1 198 151	984	40.40	1, 1, 1	38	1	6322	not published yet
New isolate 15–2067_O77	1 198 176	985	40.40	1, 1, 1	38	1	6319	not published yet
*C. abortus* S26/3	1 144 377	1002	39.87	1, 1, 1	39	1	No plasmid	NC_004552.2
*C. avium* 10DC88	1 041 170	943	36.92	1, 1, 1	39	1	7099	NZ_CP006571.1
*C. caviae* GPIC	1 173 390	990	39.22	1, 1, 1	38	1	7966	NC_003361.3
*C. felis* Fe/C-56	1 166 239	981	39.38	1, 1, 1	38	1	7552	NC_007899.1
*C. gallinacea* 08-1274/3	1 059 583	905	37.94	1, 1, 1	39	1	7619	NZ_CP015840.1
*Cand*. *C. ibidis* 10-1398/6	1 146 066	961	38.32	1, 1, 1	38	1	No plasmid	NZ_APJW00000000.1
*C. muridarum* Nigg	1 072 950	885	40.34	2, 2, 2	37	1	7501	NC_002620.2
*C. pecorum* E58	1 106 197	937	41.08	1, 1, 1	38	1	No plasmid	NC_015408.1
*C. pneumoniae* TW-183	1 225 935	1049	40.58	1, 1, 1	38	1	No plasmid	NC_005043.1
*C. psittaci* 6BC	1 171 660	984	39.06	1, 1, 1	39	1	7553	NC_015470.1
*C. suis* 1–25 a	1 088 920	902	42.03	2, 2, 2	37	1	No plasmid	NZ_FTQL00000000.1
*C. trachomatis* D/UW-3/CX	1 042 519	892	41.31	2, 2, 2	37	1	7493	NC_000117.1

### DNA extraction and hybrid sequencing of two new *Chlamydiifrater* strains

Two additional cloacal swab samples from flamingos collected in Etang du Fangassier, France, in 2015 that tested positive for *Chlamydiaceae* were successfully grown in cell culture. Subsequently, the two samples (15–2067_O09 and 15–2067_O77) were subjected to Illumina and Nanopore sequencing. DNA extraction, library preparation and Illumina and Nanopore sequencing were performed as previously described for the already published *Chlamydiifrater* strains [3]. For basecalling, we used the super-accurate model (SUP) of Guppy v6.0.0 (Oxford Nanopore Technologies). In this context, we also re-basecalled the raw signal data of the three published *Chlamydiifrater* strains for internal investigations and to double-check the results we previously obtained for fragmented *ompA* genes. Since we could not find any major differences between the assemblies generated from the original basecalls and the newly basecalled data, we decided to continue our analysis with the previously published genomes of the three original strains. This also allows an easier comparison of the results presented here with our previous study [3]. The Illumina and Nanopore raw read data for the two new strains were uploaded to EBI ENA PRJEB59173.

### Cleaning and *de novo* hybrid assembly of two newly sequenced strains

After sequencing, we decontaminated the Illumina and Nanopore sequencing data from the two new strains, 15–2067_O09 and 15–2067_O77, using CLEAN v0.2.0 (https://github.com/hoelzer/clean), a Nextflow [5] pipeline that can quickly assign reads belonging to user-defined reference sequences to sort them out. As reference sequences for decontamination, we used the complete genome of *Chlorocebus sabaeus* (Green Monkey; the cell line used to grow the isolates) and also included the mtDNA of *Chlorocebus pygerythrus* (NC_009747.1), because we discovered that even more reads can be decontaminated by adding this mitochondrial reference genome. In addition, we cleaned the Illumina reads against the PhiX phage reference and the Nanopore reads against the DCS phage reference, both commonly used control spike-ins for these sequencing technologies.

After decontamination, we quality-trimmed the Illumina reads using fastp v0.20.1 [6] (parameters: -5 -3 -W 4 -M 20 -l 15 -x -n 5 -z 6) and length-filtered the Nanopore reads using filtlong 0.2.0 (https://github.com/rrwick/Filtlong) and simultaneously downsampled to 200X coverage (based on the expected genome size of 1.2 Mbp).

Then, we assembled each genome using Unicycler v0.5.0 [7] and default parameters using the preprocessed Illumina and Nanopore data as input.

Finally, we performed independent short-read mappings of the paired-end Illumina data with BWA-MEM v0.7.17 [8], as recommended by the Polypolish manual (https://github.com/rrwick/Polypolish/wiki/How-to-run-Polypolish). We used these mappings as input for Polypolish v0.5.0 [9] to further correct potential errors in the Unicycler assemblies. The assembly quality was evaluated using QUAST v5.0.2 [10]. The final assemblies of the two new strains were utilized in all subsequent comparisons (FASTAs available at https://osf.io/dtwej).

### Reorientation of genomes

To facilitate visual genome comparison using alignment-based tools, such as Mauve [11], the circular whole-genome sequences of all 19 strains were rearranged, employing a custom Python script (located as helper script in the RIBAP repository, https://github.com/hoelzer-lab/ribap) so that the *hemB* (delta-aminolevulinic acid dehydratase) gene appears in the initial 5′ position of each genome.

### Annotation

All 17 genome sequences (excluding the outgroups) were re-annotated using Prokka v1.14.6 [12] to ensure full comparability of annotations using the same data basis. CDS with no database match were designated ‘hypothetical protein’. The annotation was performed twice. Prokka was used with default parameters in the first pass, mainly using the UniProtKB database for functional protein annotation. In the second pass, a GenBank annotation file downloaded from NCBI was additionally provided via the parameter --proteins for each genome sequence as an initial proxy for functional annotation assignments (Table S1). The --proteins option is recommended to ensure consistent gene naming, particularly for species using specific terminology [12].

For the chlamydial genome sequences included in this study, the number of CDS annotated as hypothetical proteins in the first and second runs was gathered and compared. Since the number of hypothetical proteins was lower in the second run, and Prokka is an integral part of RIBAP, which we used for the pan-genome calculations, we decided to continue our downstream analysis with these harmonized and extended Prokka annotations.

### Roary ILP Bacterial Annotation Pipeline

We used the Roary ILP Bacterial Annotation Pipeline (RIBAP) for comparative genome analysis and core gene set calculations [4]. RIBAP is freely available at https://github.com/hoelzer-lab/ribap and is implemented using the workflow management system Nextflow [5].

Each pipeline step is encapsulated in its own Docker container [13]. Thus, only Nextflow and Docker are required to run RIBAP. For the present study, the pipeline was run in v0.7.1 using the following command:

nextflow run hoelzer-lab/ribap -r 0.7.1 --list --fasta ribap-input.csv --reference ribap-ref.csv -w work -profile local,docker

The parameter --fasta is used to feed a CSV file into the pipeline containing a list of the paths to the input genome sequences. The CSV file, passed to RIBAP via the --reference parameter, contains the path to one NCBI GenBank file for each of these FASTA files to be used as additional reference during Prokka v1.14.6 [12] annotation (Table S1). We use these additional reference annotation files to improve the functional re-annotation by Prokka. In addition, the re-annotation ensures comparable analyses. The subsequent core genome calculation is based on Prokka’s CDS annotations, including hypothetical proteins. All input files, intermediate files and complete results of the pipeline are provided at https://osf.io/dtwej.

We executed the pipeline twice: first, on the 12 *Chlamydia* species, 3 *Chlamydiifrater* strains and the 2 newly assembled flamingo isolates, and second, on the full set of all 19 genomes included in this study (comprising 2 additional outgroups, *W. chondrophila* 86–1044 and *S. negevensis* Z). The 17 genomes from the first run and their orthologous gene groups identified with RIBAP were used as input for UpSetR (v1.4.0) and all general pan-genome investigations. The UpSet visualization of all 17 strains was restricted to the largest 40 intersecting sets. The core genome of the 19 strains from the second pass was used for calculating a phylogenetic tree. RIBAP utilizes MAFFT v7.455 [14] to create a multiple sequence alignment (MSA) for each RIBAP group and concatenates the resulting alignments. The concatenated MSA was used to construct a phylogenetic maximum likelihood tree using a bootstrap value of 100 and the LG+G4 substitution model with IQ-Tree v2.2.0-beta [15,16].

### Visual comparison of genome sequences

We used Mauve v2.4.0.r4736 [11] for whole-genome comparison and aligned the genomes of *Chlamydia psittaci* 6BC, *Cf. phoenicopteri* 14–2711_R47 (R47), *Cf. volucris* 15–2067_O50 (O50) and 15–2067_O99 (O99), as well as the new isolates 15–2067_O09 (O09) and 15–2067_O77 (O77). The MSA was constructed using progressive Mauve. We used the tool’s visualization feature to display the rearrangement structure of the genome sequences.

## Results

### Refinement of genome sequences prior to analysis

This study includes a total of 17 strains, comprising 2 new flamingo isolates which were sequenced and assembled (15–2067_O09, 15–2067_O77) and 3 previously published strains: *Cf. phoenicopteri* 14–2711_R47, *Cf. volucris* 15–2067_O50 and 15–2067_O99. All *Cf. volucris* strains were obtained during the same sampling campaign. Furthermore, we included genome sequences of type or reference strains of 12 *Chlamydia* spp. for comparative analysis. For phylogenetic analysis, we additionally used *W. chondrophila* 86–1044 and *S. negevensis* Z as outgroups.

**Fig. 1.**
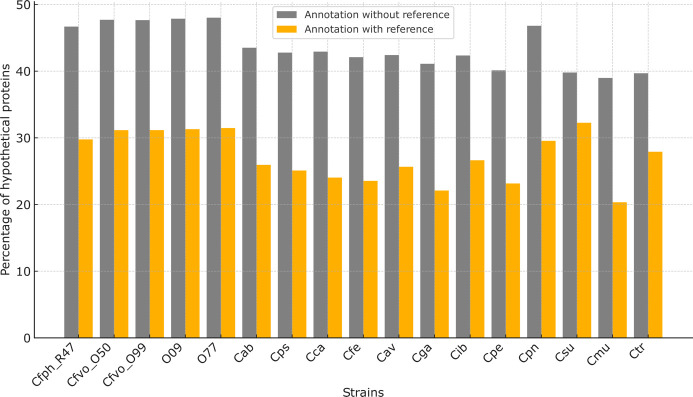
The percentage of hypothetical proteins after annotation with Prokka in relation to the total number of annotated CDS. Grey bars show the percentage of hypothetical proteins using Prokka with default parameters. In contrast, orange bars show results using additional reference annotations to guide the functional annotation process. Additional references used for each strain are provided in Table S1. Cfph, *Chlamydiifrater phoenicopteri*; Cfvo, *Chlamydiifrater volucris*; Cab, *Chlamydia abortus*; Cps, *Chlamydia psittaci*; Cca, *Chlamydia caviae*; Cfe, *Chlamydia felis*; Cav, *Chlamydia avium*; Cga, *Chlamydia gallinacea*; Cib, *Candidatus Chlamydia ibidis*; Cpe, *Chlamydia pecorum*; Cpn, *Chlamydia pneumoniae*; Csu, *Chlamydia suis*; Cmu, *Chlamydia muridarum*; Ctr, *Chlamydia trachomatis*.

We strove to eliminate potential technical bias due to different annotation tools, reference databases and parameter settings to allow adequate comparative analysis. Therefore, we re-annotated all genomes using Prokka [12], which assigned only 52–61% of the CDS a specific function. In contrast, the remaining 39–48% were still at ‘hypothetical protein’ status. To further improve the functional annotation and specifically reduce the number of hypothetical proteins, we ran Prokka for each genome with a matching reference annotation from NCBI (Table S1). This enabled us to re-annotate all genomes using reference annotations, search for new CDS and increase the degree of harmonization in downstream analysis. With these additional reference annotations for Prokka runs, we could assign 12–19% more CDS to a specific function, thereby reducing the proportion of hypothetical proteins to only 20–32%. The bar chart in Fig. 1 shows the percentage of hypothetical proteins (in relation to the total number of CDS) after annotation with and without reference genome information using the same annotation software. Apart from the larger genome of *Chlamydia pneumoniae* and the smaller genome of *Chlamydia suis*, the *Chlamydiifrater* genomes still contain the highest number of hypothetical proteins, which had to be expected because these organisms were only recently discovered.

### General characteristics

The basic parameters of the present panel of 17 strains are given in Table 1. With genome sizes between 1 195 517 and 1 198 895 bp, *Chlamydiifrater* strains were found to have slightly larger genomes than *Chlamydia* spp., except *C. pneumoniae* (1 225 935 bp).

**Fig. 2.**
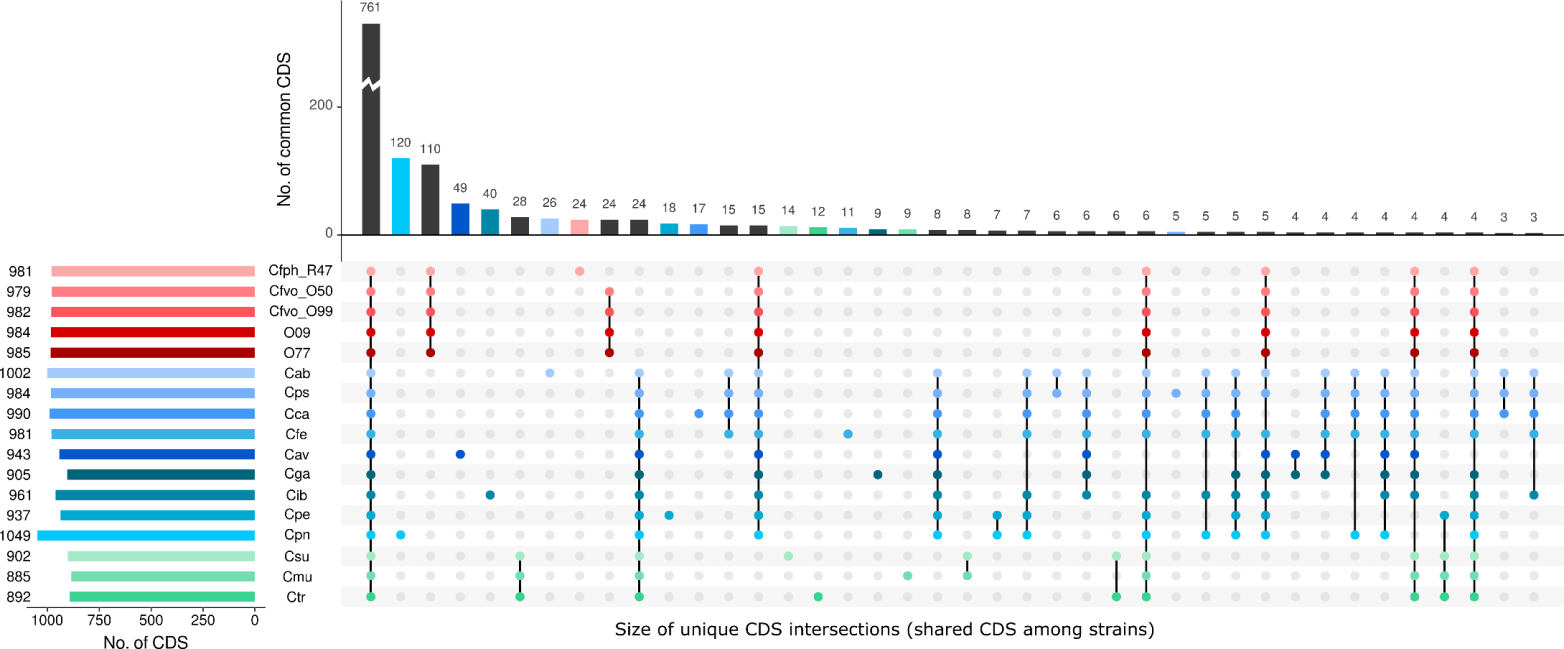
UpSet bar diagram showing the number of genes shared by various groups of chlamydial strains selected for this study. Calculations are based on whole-genome sequences annotated with Prokka of the 3 published *Chlamydiifrater* strains, the 2 newly assembled strains 15–2067_O09 and 15–2067_O77 and type or reference strains of 12 members of the genus *Chlamydia*. Orthologous groups were calculated using the RIBAP pipeline and used as input for UpSetR. Cfph, *Chlamydiifrater phoenicopteri*; Cfvo, *Chlamydiifrater volucris*; Cab, *Chlamydia abortus*; Cps, *Chlamydia psittaci*; Cca, *Chlamydia caviae*; Cfe, *Chlamydia felis*; Cav, *Chlamydia avium*; Cga, *Chlamydia gallinacea*; Cib, *Candidatus Chlamydia ibidis*; Cpe, *Chlamydia pecorum*; Cpn, *Chlamydia pneumoniae*; Csu, *Chlamydia suis*; Cmu, *Chlamydia muridarum*; Ctr, *Chlamydia trachomatis*.

The results of core genome calculation using RIBAP and comparative analysis using UpSetR are shown in Fig. 2. The number of CDS per genome ranges from 885 (*Chlamydia muridarum* Nigg) to 1049 (*C. pneumoniae* TW-183). A total of 761 CDS are shared among members of both genera. On the other hand, we detected 110 genus-specific genes and an additional 24 genes only in either *Cf. volucris* or *Cf. phoenicopteri* strains. Therefore, in these five strains, 16.1% of the total coding capacity (average no. of CDS 982) consists of entities unique to members of the genus *Chlamydiifrater*.

**Fig. 3.**
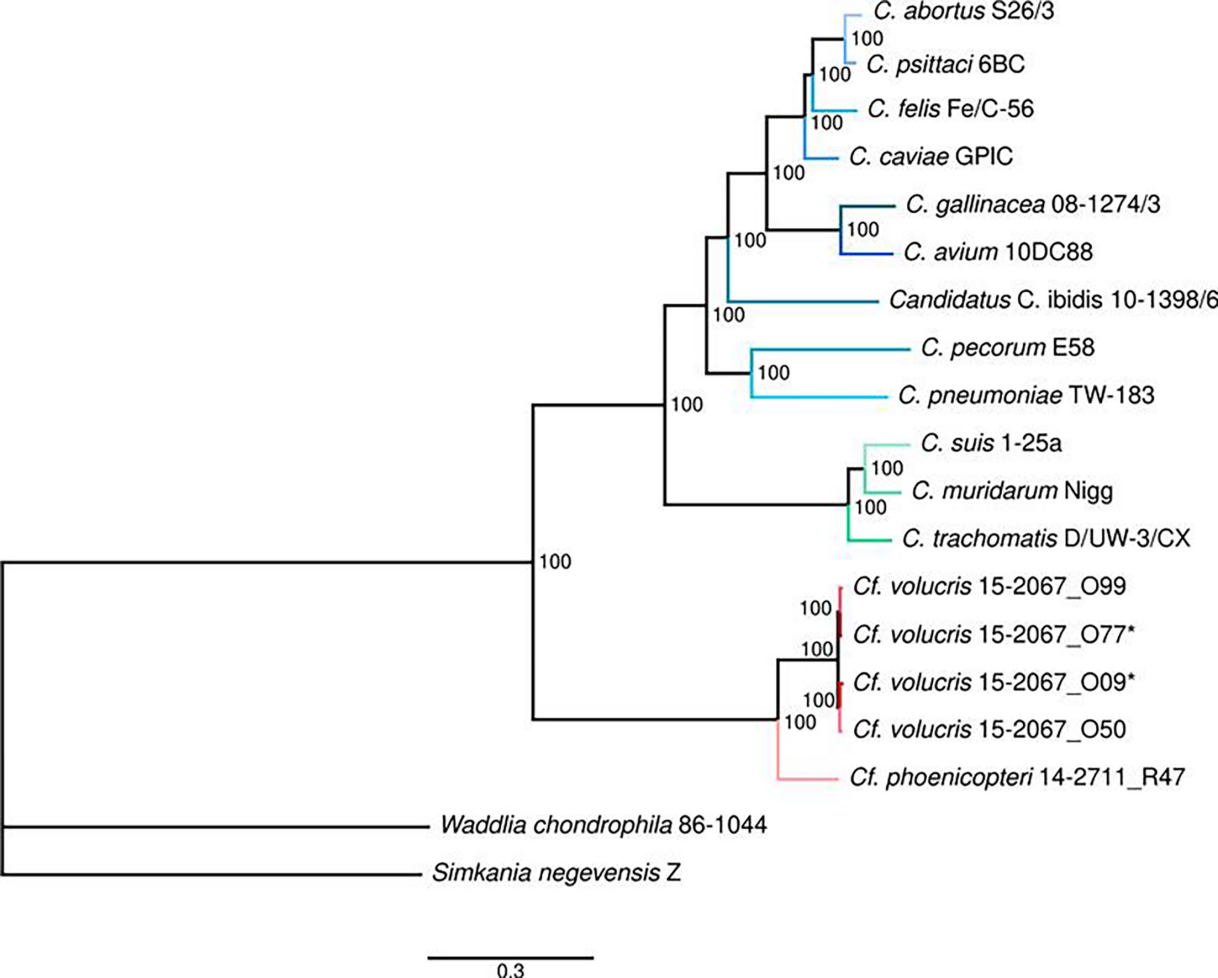
Phylogenetic tree of all strains of *Chlamydia* and *Chlamydiifrater* included in this study. Strains marked by an asterisk were newly sequenced and assembled. The phylogeny was reconstructed based on the concatenated MSA of the core genomes predicted by RIBAP. The tree was constructed using IQ-Tree with LG+G4 substitution model. Bootstrap values are shown as percentage and indicate the stability of the branches based on 1000 replicates. The scale bar shows the number of amino acid substitutions per site. Chlamydial strains are coloured as in Fig. 2. *W. chondrophila* 86–1044 and *S. negevensis* Z were used as outgroups.

To characterize phylogenetic relationships of the five *Chlamydiifrater* strains with the species of the genus *Chlamydia*, a phylogenetic tree was reconstructed based on a multiple sequence alignment of the concatenated core genes. The tree in Fig. 3 confirms the separate position of members of the genus *Chlamydiifrater* and the classification of the newly sequenced strains 15–2067_O09 and 15–2067_O77 as belonging to the species *Cf. volucris*.

### Search for genomic loci belonging to the chlamydial plasticity zone

Since the PZ in *Chlamydia* genomes can be defined as the region between *accB* (5′-terminus) and *guaB* genes (3′-terminus), we conducted blast searches using the genes annotated in the PZ of *C. psittaci* 6BC as queries [17]. However, we were only able to identify *accB* (encoding biotin carboxyl carrier protein of acetyl-CoA carboxylase), *accC* (biotin carboxylase) and *toxB* (LifA/Efa1-related large cytotoxin or large cytotoxin or lymphostatin/EFA-1 ortholog) in the *Chlamydiifrater* genomes. We found no orthologs of a gene for the membrane attack complex/perforin (MACPF), one of the major elements in the PZ of *C. psittaci, Chlamydia felis, Chlamydia pecorum, Chlamydia trachomatis, C. suis* and *C. muridarum*. Likewise, there was no evidence of a tryptophan operon as encountered in *C. trachomatis, C. suis, Chlamydia caviae* and *C. felis*.

In addition, genes of the purine synthesis operon *guaA* (GMP synthase), *guaB* (inosine-5′-monophosphate dehydrogenase) and ADA (adenosine/AMP deaminase), which are indicative of the PZ 3′-terminus in *Chlamydia* spp., were also missing in the *Chlamydiifrater* genomes. Characteristic features of the PZ in strains of *Chlamydiifrater* and *Chlamydia* are summarized in Table 2.

**Table 2.** Plasticity zone parameters of *Chlamydiifrater* strains and representative strains of *Chlamydia* spp.

Species strain	PZ total size (nt)	# CDS	Biotin modification	*toxB* (nt)	SSS (nt)	MAC/ perforin†(nt)	Trp operon	Purine synthesis and recycling
*Cf. phoenicopteri*14–2711_R47	38 200	21	*accB, accC*	9870	1836 + 1836*	–	–	–
*Cf. volucris*15–2067_O50	38 200	19	*accB, accC*	9885	1455	–	–	–
*Cf. volucris*15–2067_O99	38 200	19	*accB, accC*	9876	1455	–	–	–
New isolate*Cf. volucris*15–2067_O09	38 200	19	*accB, accC*	9903	1455	–	–	–
New isolate*Cf. volucris*15–2067_O77	38 200	19	*accB, accC*	9888	1455	–	–	–
*C. caviae* GPIC	34 753	21	*accB, accC*	10041	–	(504)‡	*trpA, trpB_1/2, trpD, trpF, trpR, kynU*	*guaA, guaB*, ADA
*C. felis* Fe-C56	39 924	28	*accB, accC*	9897	–	2442; (501)	*trpA, trpB, trpD, trpF, trpR, kynU*	*guaA, guaB*, ADA
*C. pneumoniae* TW-183	8759	11	*accB, accC*	–	–	1236	–	*guaB_1/2*‡
*C. psittaci* 6BC	29 145	16	*accB, accC*	10074	–	2469; (627)	–	*guaA, guaB*, ADA
*C. trachomatis* d-UW-3-CX	55 445	49	*accB, accC*	–	–	2433	*trpA, trpB, trpR*	–

*Two different ORFs of the same size (hypothetical proteins) instead of SSS (sodium solute symporter family protein).

†In parenthesis: size (nt) of the gene coding for MAC/perforin domain-containing protein.

‡Pseudogenes.

*accB*, biotin carboxyl carrier protein of acetyl-CoA carboxylase; *accC*, biotin carboxylase.; ADA, adenosine/AMP deaminase; *guaA*, GMP synthase; *guaB*, inosine-5′-monophosphate dehydrogenase.

**Fig. 4.**
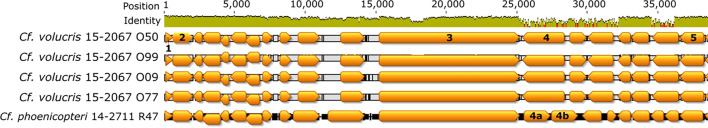
Alignment of the PZ of *Chlamydiifrater* strains. (1) *accB*, (2) *accC*, (3) large cytotoxin, (4) SSS family protein, (4a, 4b) DUF1389 domain-containing protein (*Cf. phoenicopteri* only), (5) DUF648 domain-containing protein.

The alignment of the genomic segments harbouring the putative PZ is schematically depicted in Fig. 4. Structural differences between the species of *Cf. phoenicopteri* and *Cf. volucris* can be observed in the segment from *accB/accC* (pos. 7,238) to ORF10 (pos. 48,627), thus covering a region of ~41 kbp. The *accB* gene assumed to represent the PZ’s 5′-terminus is preceded by the gene coding for ribulose-phosphate 3-epimerase. Moving in the 3′ direction, nine minor ORFs common to all five strains are encountered. In the central PZ region, the *toxB* gene encoding the LifA/Efa1-related large cytotoxin ortholog is the dominant element. Sequences of this gene are quite variable both between the two species and among the *Cf. volucris* strains as well.

**Fig. 5.**
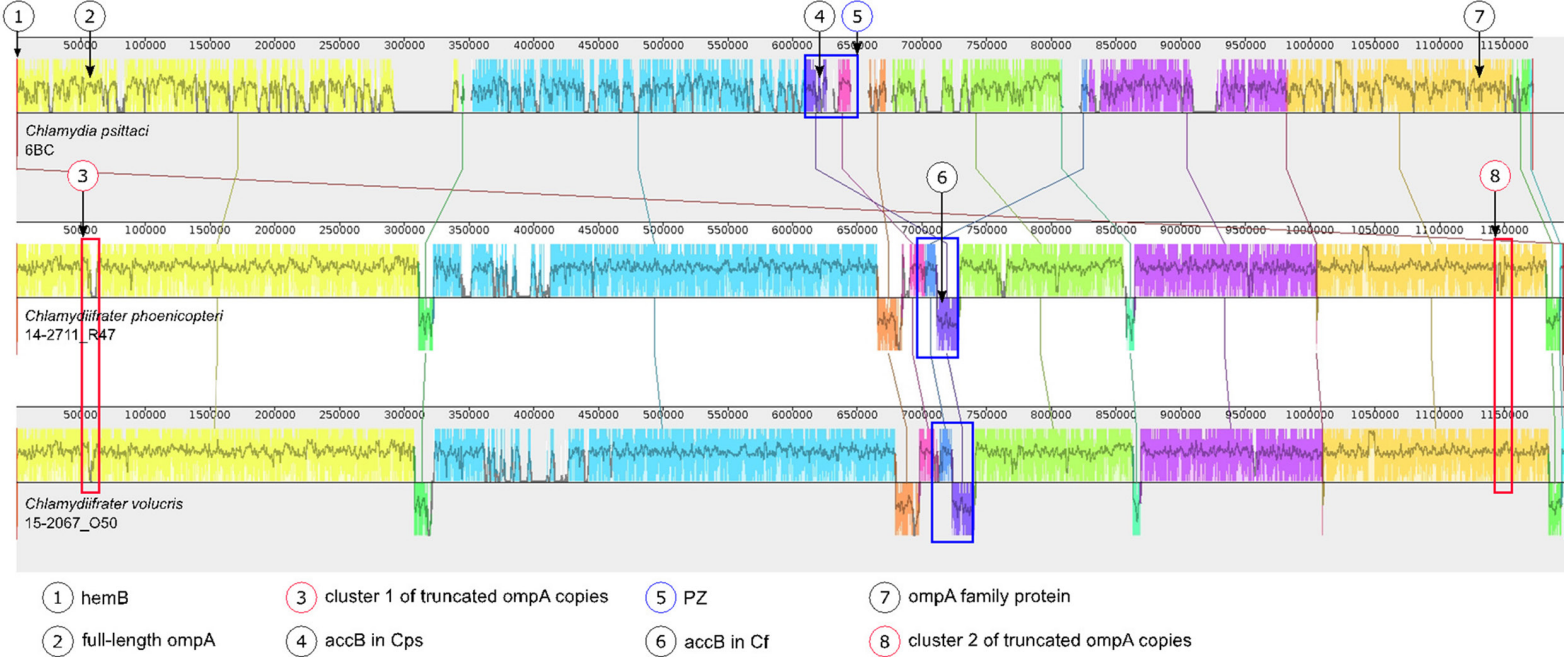
Genome alignment of whole-genome sequences of *C. psittaci* 6BC and type strains of *Cf. phoenicopteri* and *Cf. volucris*. All genomes were rearranged to have the gene *hemB* at the 5′-end. The arrows above the figure indicate the approximate position of the following gene loci: full-length *ompA*, *ompA* family protein and *accB*, the 5-terminal ORF of the PZ. The three gene loci are present at a similar position in all six sequences. In addition, areas in the genomes of the *Chlamydiifrater* strains where truncated *ompA*-like porins and typical genes for the chlamydial PZ were found are highlighted. The multiple genome alignment and figure were created with progressive Mauve and edited using Inkscape. Details of the full-length *ompA*, *ompA* family proteins and the truncated *ompA*-like porins are shown in Fig. 6.

The PZ region 3′ of *toxB* is the most heterogeneous among the strains. It contains a CDS encoding a sodium solute symporter (SSS) family protein, whose sequence varies from strain to strain in *Cf. volucris* and is replaced in *Cf. phoenicopteri* with two smaller ORFs. Further downstream, the next five ORFs in *Cf. volucris* are hypothetical proteins, followed by the DUF648 domain-containing protein, which is already less variable. This ORF probably indicates the 3′-end of the PZ since the differences between the species and strains decrease further downstream. In *Cf. phoenicopteri*, the same region between toxin and DUF648 contains eight smaller ORFs. Overall, the PZ size in this genus is around 38 200 nt. The location of the putative PZ region is shown in an alignment of whole-genome sequences in Fig. 5.

### Loci encoding *ompA* and members of the *ompA* family

Four *Chlamydiifrater* genomes were found to harbour a full-length (1179 to 1206 nt) *ompA* sequence encoding the porin OmpA of 393 to 402 aa, which proved orthologous to counterparts in *Chlamydia* spp. Alignment of translated proteins revealed a pattern of four variable domains as known from *Chlamydia* spp. As an exception, the *ompA* of *Cf. volucris* strain O09 seemed to be shorter, i.e. 376 aa. However, as we will explain in the ‘Discussion’ section, this is an assembly artefact.

**Fig. 6.**
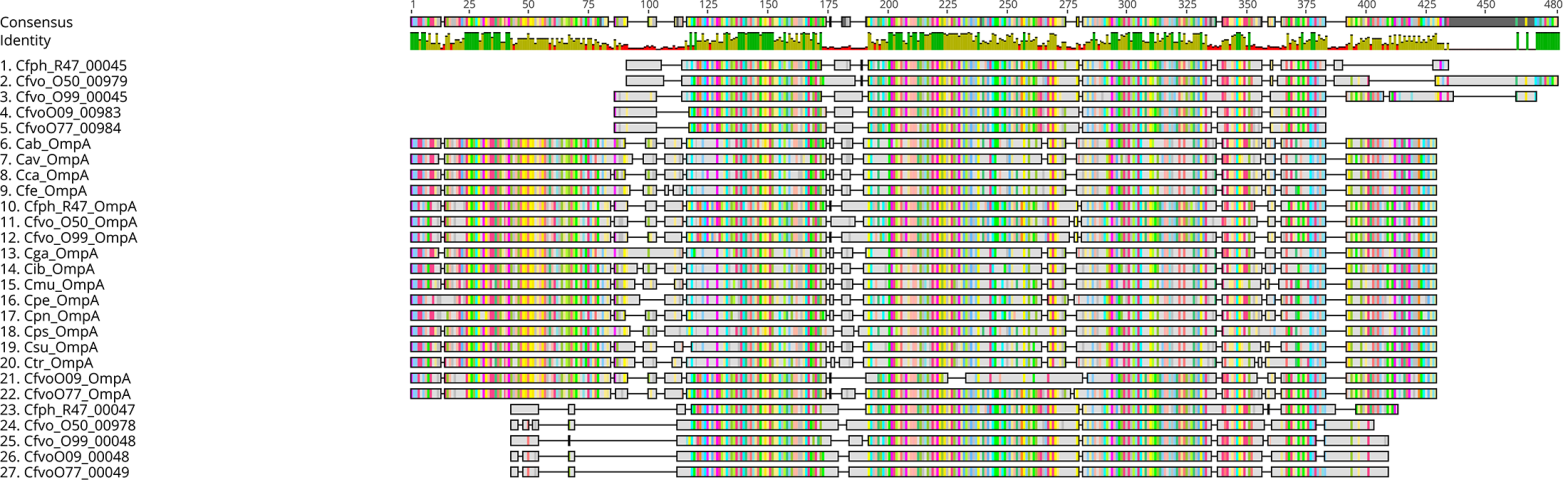
Schematic depiction of the multiple amino acid alignment of (i) full-length OmpA/MOMP proteins of *5 Chlamydiifrater* and 12 *Chlamydia* strains (lines 6–22), (ii) OmpA family proteins of 5 *Chlamydiifrater* strains (RIBAP group 930.2, lines 1–5) and (iii) another set of truncated family members (RIBAP group 930.3, lines 23–27). The colours are randomly assigned by the Geneious software used for visualization to illustrate homologous sequence regions. Cfph, *Chlamydiifrater phoenicopteri*; Cfvo, *Chlamydiifrater volucris*; Cab, *Chlamydia abortus*; Cps, *Chlamydia psittaci*; Cca, *Chlamydia caviae*; Cfe, *Chlamydia felis*; Cav, *Chlamydia avium*; Cga, *Chlamydia gallinacea*; Cib, *Candidatus Chlamydia ibidis*; Cpe, *Chlamydia pecorum*; Cpn, *Chlamydia pneumoniae*; Csu, *Chlamydia suis*; Cmu, *Chlamydia muridarum*; Ctr, *Chlamydia trachomatis*.

In addition, we identified several truncated *ompA* family members aligning partially to full-length *ompA* (Fig. 6). To rule out the possibility that these truncated CDS might be due to sequencing or assembly artefacts, we re-basecalled the original raw signal FAST5 data of previously published *Chlamydiifrater* genomes and the two new sequencing runs using the latest versions of Guppy (version 6) and the SUP model. These sequences were subsequently combined with the corresponding Illumina data to be re-assembled using Unicycler v0.5.0 [40]. This confirmed the previously observed structural pattern consisting of (i) one full-length version of the *ompA* gene, (ii) a cluster of truncated *ompA* copies, (iii) another cluster of (truncated) *ompA*-like porin genes [3] and (iv) an additional family member annotated as ‘*ompA* family protein’, which could not be unambiguously assigned. It is noteworthy that the present assembly of *ompA* clusters and individual genes is supported by long-read data.

The locations of the *ompA* family members are shown in a Mauve alignment of whole-genome sequences in Fig. 5. The truncated family members, annotated as ‘porin’, are distributed throughout all five *Chlamydiifrater* genomes according to the same pattern. They are arranged in two clusters, and their numbers vary from strain to strain: 15 in *Cf. phoenicopteri* R47 and in *Cf. volucris*, 5 in strain O50, 9 in strain O99 and 8 in both O09 and O77. Fig. 6 shows a schematic depiction of the multiple amino acid alignment of the full-length *ompA*, *ompA* family proteins and the truncated *ompA*-like porins highlighted in Fig. 5.

### Virulence genes known from *Chlamydia* spp*.*

Next, we wanted to check whether some of the already known putative virulence genes and other prominent factors from *Chlamydia* spp. are present in members of the genus *Chlamydiifrater*. This is the case with the gene encoding chlamydia protease-like activity factor, which was retrieved in all five *Chlamydiifrater* strains (RIBAP group 713). Also, *uvr*ABC genes, which encode a multienzyme complex involved in DNA repair, were encountered in all *Chlamydiifrater* strains.

Our analysis also identified six genes encoding members of the Fts protein family, all of which are shared with *Chlamydia* spp. genomes. They include FtsH (RIBAP group 728), FtsK (811), FtsW (792), FtsX (559), FtsY (747) and an FtsW/RodA/SpoVE family cell cycle protein (821).

Analogous to *Chlamydia*, *Chlamydiifrater* strains also seem to possess a type III secretion system (T3SS) as 24 genes were annotated accordingly, among them SctD, E, J, N, Q, R, S, U, V and W, as well as actin-recruiting effector Tarp.

In contrast, members of the autotransporter or polymorphic membrane protein (Pmp) and inclusion protein (Inc) families could not be found. Likewise, several presumed chlamydial virulence factors, such as Cadd, SinC and HctA/B, were not seen in *Chlamydiifrater*.

### Search for *Chlamydiifrater*-specific genes

The UpSet plot in Fig. 2 generated from the output of the RIBAP pipeline revealed 110 CDS that are unique for the genus *Chlamydiifrater* and that are shared among all 5 strains. The RIBAP pipeline output also showed a total of 110 CDS occurring in the 5 *Chlamydiifrater* strains, but not in the *Chlamydia* strains. While the vast majority of these genes have eluded functional annotation, the few annotated genes include orotidine 5′-phosphate decarboxylase, NAD(+)/NADH kinase, RluA family pseudouridine synthase, SDR family NAD(P)-dependent oxidoreductase, 3′−5′ exonuclease, chromosome partition protein gene *smc* and seven genes of truncated porins (RIBAP groups 930.1 to 930.7; see https://osf.io/dtwej for complete output).

We also observed 24 CDS unique to *Cf. phoenicopteri* R47 (Fig. 2), among them 7 (truncated) porins and orotate phosphoribosyltransferase *pyrE*. Of the 24 unique genes of the 4 *Cf. volucris* strains, only one was annotated (helix-turn-helix domain-containing protein), while the rest remained as ‘hypothetical proteins’.

## Discussion

### Re-annotation provides extended insights into the organization of the *Chlamydiifrater* genome

With a size of ~1.2 Mbp, the *Chlamydiifrater* spp. have larger genomes than *Chlamydia* spp., except for *C. pneumoniae*. We performed re-annotation to facilitate the comparison of the various genome sequences in our study and to reduce the impact of technical differences between the annotation tools and versions used. After an initial re-annotation with default parameters, we found many hypothetical genes with no assigned function. Therefore, we decided to improve functional gene annotation by adding additional reference information to the annotation process. For each genome included, we also obtained the annotation of the corresponding reference strain or a closely related strain from NCBI GenBank (Table S1). We included this information to improve functional gene assignments, which resulted in a lower number of hypothetical genes overall and a harmonized annotation dataset for downstream analysis. In general, for rapid gene annotation using a reference database of limited size (e.g. UniProtKB, a core database in Prokka), we recommend adding additional reference information when available to improve functional annotation and ensure consistent gene naming.

### Deciphering the plasticity zone to unveil novel genomic landscapes and diversification strategies in *Chlamydiifrater* spp.

The PZ has drawn the interest of genome researchers, because it is one of the few genomic regions with lower synteny in *Chlamydia* spp. Otherwise, the relatively small genomes of these species are highly conserved in gene content and order and, consequently, also their metabolic capacities [18,19]. Therefore, it was hypothesized that analysis of the PZ might provide a clue to understanding the significant differences among individual species of *Chlamydia* in terms of tissue tropism, host preference, immune response patterns and pathogenicity [20]. A recent study analysing genome sequences of 61 *C*. *psittaci* strains indicated that the absence of MACPF in the PZ could be associated with different host preferences of the respective strains [17].

Concerning *Chlamydiifrater* spp. genomes, it was not known previously whether they harboured a PZ and, if so, where it was located and which genes it encompassed. Our study is the first to confirm that typical elements known from the 5′-terminal PZ region in *Chlamydia* spp. are present. At the same time, the segment downstream of the *toxB* analogue until the 3′-terminus is distinct from the chlamydial counterpart. In addition, the absence of a *guaAB*-ADA locus at the 3′-terminus of the *Chlamydiifrater* PZ might indicate a different purine salvage strategy [21].

### *OmpA* sequences, deletions in *ompA*-like CDS and possible structural consequences

We observed a low sequence similarity of *Chlamydiifrater* full-length *ompA* genes to their counterparts in *Chlamydia*. While values from 65 to 85% are typical among species of the latter genus, *ompA* genes of *Chlamydiifrater* strains showed values below 56% (Table S2).

Therefore, 3D structures are likely to differ significantly between the two genera. We previously demonstrated that, in *C. psittaci*, deletions of a few amino acids in variable domains 1, 2 and 4 already caused structural changes capable of changing surface antigen properties from one strain to another [17].

As mentioned above, during our analysis of the *ompA* locus across the different *Chlamydiifrater* strains, a notable exception was observed with strain O09. Unlike other strains harbouring a full-length *ompA* gene, strain O09 presented only fragmented sequences in this genomic region. Detailed bioinformatic analysis identified the *ompA* sequence assembled and annotated in three separate ORFs spanning positions 67 426 to 68 595 on the minus strand of the rearranged O09 genome. This fragmentation appeared to result from sequencing and assembly challenges, as confirmed by additional alignments and annotations using Prokka and Bakta [22], and could not be rectified through additional long-read-only assembly using Flye [23]. Thus, the putative *ompA* gene in O09 would be about 1169 nt in size, corresponding to about 389 aa, but with some uncertainty due to incomplete assembly in the genome. The *ompA* of strain O77, which displayed no such fragmentation, was used as a reference to identify a complete *ompA* sequence in O09, revealing alignment over a nearly identical region. This suggests that the observed fragmentation in O09 may be attributed to issues in sequencing fidelity and assembly rather than genetic divergence. A higher sequencing depth or longer read lengths in this region and additional Sanger sequencing could be used to close this gap in the assembly and improve the annotation. Another possibility could be to re-basecall the raw nanopore signal data using newer models and then perform *de novo* assembly again to check if a fully assembled *ompA* locus can be achieved in O09. To circumvent these issues and enable meaningful comparisons within the RIBAP group 930, we manually constructed a putative full-length *ompA* sequence from the three fragmented ORFs found by Prokka, incorporating artificial sequences to indicate the junctions. This reconstructed sequence allows O09 to be included in multi-strain analysis despite the underlying assembly challenges. In this context, also note that RIBAP had assigned one of the (truncated) *ompA*-like sequences of strain O09 to the group 930 because it better matched than any of those three fragmented *ompA* ORFs belonging to full-length *ompA* of O09. While the presence of a full-length *ompA* gene encoding MOMP, homologous to its counterpart in *Chlamydia* spp., could be expected, detecting two clusters of truncated family members was a novel observation in our previous study [3]. In this study, we took a closer look at the sequences of the *ompA*-like CDS. As a common feature, they all lack the conserved N-terminal domain of the full-length protein. For example, Fig. 6 shows that up to 90 aa can be missing compared to full-length MOMP. The N-terminal domain contains the signal peptide (1–22 aa), which means that if the truncated CDS possesses some functionality, it would differ from that of MOMP. At least their integration into the outer membrane of *Chlamydiifrater* may be impossible. In addition, the C-terminal part of up to 40 aa was also absent in most truncated copies (Fig. 6).

### Unique *Chlamydiifrater genes* and the absence of certain genes encountered in *Chlamydia* spp.

To understand the distinct traits and unique characteristics of *Chlamydiifrater* organisms, we sought to identify genomic elements not encountered in their closest relatives, *Chlamydia* spp. The finding that 16.1% of *Chlamydiifrater* genes are present only in the strains of this genus raises the question of their significance and possible functions. In fact, these genome sequences could encode products that define unique properties distinguishing *Chlamydiifrater* from *Chlamydia* species and other bacteria.

On the one hand, there are several essential cellular genes and potential virulence genes already known from *Chlamydia* spp. that are also present in *Chlamydiifrater*. For instance, members of the Fts protein family, part of the bacterial divisome, are a complex and highly dynamic molecular machinery with more than 20 components [24]. FtsZ, one of the best-studied proteins of the complex, is absent in both *Chlamydiifrater* and *Chlamydia* spp. While FtsK and FtsW are essential factors for cell division [25,26], the functionalities of FtsH, FtsX and FtsY are still unknown. Our findings suggest that the cell division machinery in *Chlamydiifrater* is similar to that of *Chlamydia* organisms, but different from that of many other bacteria, e.g. *Escherichia coli*.

The identification of a T3SS in our analysis also represents an important finding. This needle-like protein complex, formed by ~30 proteins, is used by the bacterium to secrete certain effector proteins into the host cell in the context of colonization and/or pathogenesis. Given the 24 genes identified here, it can be assumed that the *Chlamydiifrater* organisms harbour a complete and functional T3SS.

On the other hand, the considerable proportion of CDS unique to *Chlamydiifrater* is noteworthy. However, as most CDS elements eluded automatic annotation due to a lack of functional data in public databases, it is difficult to draw conclusions. blast analysis of amino acid sequences of unique proteins in *Cf. phoenicopteri* or *Cf. volucris*, respectively, failed to retrieve orthologs in other organisms, so no indications of possible functions could be obtained.

## Conclusions

This study extends the number of publicly available *Chlamydiifrater* genomes and represents a comprehensive genomic study of the detailed landscape of their genetic makeup. Our data revealed several genomic features distinguishing them from the closely related *Chlamydia* species. Identifying several truncated CDS of *ompA*/MOMP and the presence of a *Chlamydia*-like plasticity zone indicates the unique evolutionary pathway of the genus *Chlamydiifrater*. Although species of *Chlamydiifrater* share an extensive core genome with those of *Chlamydia*, the high proportion of genus-specific genes suggests a unique repertoire of functions and adaptations potentially relevant to their pathogenicity and host interactions. The advanced bioinformatic workflows and hybrid sequencing techniques used here have helped to refine our understanding of these recently discovered bacteria. However, the example of the fragmented *ompA* gene in strain O09 shows how important it is to scrutinize individual genomic results, even at the age of long-read sequencing and advanced computational genomics, as sequencing and assembly can still be challenging. Finally, the genetic novelties discovered and described here not only contribute valuable data to the field of microbial genomics but also pave the way for future research into the biology, infection mechanisms and potential treatment of disease.

## Supplementary material

10.1099/acmi.0.000936.v3 Uncited Table S1. and S2

## Abbreviations

EBs: elementary bodies
MACPF: membrane attack complex/perforin
MOMP: major outer membrane porin
MSA: multiple sequence alignment
NCBI: National Center for Biotechnology Information
PZ: plasticity zone
RBs: reticulate bodies
RIBAP: Roary ILP Bacterial Annotation Pipeline
SSS: sodium solute symporter
SUP: super-accuracy
T3SS: type III secretion system
